# Regulating Effect of Exogenous α-Ketoglutarate on Ammonium Assimilation in Poplar

**DOI:** 10.3390/molecules29071425

**Published:** 2024-03-22

**Authors:** Xiaoning Liu, Liangdan Wu, Yujia Si, Yujie Zhai, Mingyi Niu, Mei Han, Tao Su

**Affiliations:** 1 Co-Innovation Center for Sustainable Forestry in Southern China, College of Life Sciences, Nanjing Forestry University, Nanjing 210037, Chinayujiezhai@163.com (Y.Z.);; 2 Key Laboratory of State Forestry Administration on Subtropical Forest Biodiversity Conservation, Nanjing Forestry University, Nanjing 210037, China

**Keywords:** ammonium, poplar, α-ketoglutarate, growth promotion

## Abstract

Extensive industrial activities and anthropogenic agricultural practices have led to substantial ammonia release to the environment. Although croplands can act as ammonia sinks, reduced crop production under high concentrations of ammonium has been documented. Alpha-ketoglutarate (AKG) is a critical carbon source, displaying pleiotropic physiological functions. The objective of the present study is to disclose the potential of AKG to enhance ammonium assimilation in poplars. It showed that AKG application substantially boosted the height, biomass, and photosynthesis activity of poplars exposed to excessive ammonium. AKG also enhanced the activities of key enzymes involved in nitrogen assimilation: glutamine synthetase (GS) and glutamate synthase (GOGAT), elevating the content of amino acids, sucrose, and the tricarboxylic acid cycle (TCA) metabolites. Furthermore, AKG positively modulated key genes tied to glucose metabolism and ATP synthesis, while suppressing ATP-depleting genes. Correspondingly, both H^+^-ATPase activity and ATP content increased. These findings demonstrate that exogenously applying AKG improves poplar growth under a high level of ammonium treatment. AKG might function through sufficient carbon investment, which enhances the carbon–nitrogen balance and energy stability in poplars, promoting ammonium assimilation at high doses of ammonium. Our study provides novel insight into AKG’s role in improving poplar growth in response to excess ammonia exposure.

## 1. Introduction

Nitrogen is a vital nutrient for plant growth, aiding in synthesizing proteins, amino acids, nucleic acids, chlorophyll, and various metabolites that drive photosynthesis and carbohydrate production [1]. In modern agriculture, nitrogen-based fertilizers, typically nitrates and ammonium, have significantly boosted crop yields over recent decades. However, the intense application of nitrogen fertilizers has led to environmental degradation, including considerable ammonia emissions that impair air, soil, and water quality and harm biodiversity [2,3]. Besides, ammonia also takes part in producing fine particulate matter (PM_2.5_), posing risks to the health of human beings [2]. Hence, it is crucial to balance the benefits of ammonia in agriculture with its environmental footprint, ensuring sustainable farming practices that protect crop productivity and ecological health.

At an ammonia compensation point, plants can act as natural filters for ammonia, assimilating ammonia–nitrogen into organic forms to support their growth and yield [4,5]. A few species, such as *Spartina alterniflora*, rice, and citrus can use ammonium as the predominant nitrogen source [6]. Nonetheless, for most plant species, excessive ammonium (NH_4_^+^) in the soil can be detrimental [4,7], resulting in root medium acidification, a decline in photosynthesis, disruptions in cytoplasmic pH, carbohydrate shortages, and imbalances in carbon and nitrogen metabolism [8,9,10]. These interruptions can hinder energy efficiency within the plant [11,12], and may cause leaf chlorosis, biomass reduction, or potentially plant death [4]. Thus, the questions how to avoid the acidification of matrix in an ammonium-enriched environment for plants and how to prevent nitrification in conditions where ammonium becomes the predominant nitrogen source need to be addressed.

Alpha-ketoglutarate (AKG) is a crucial compound in cellular metabolism, known as 2-ketoglutarate, 2-oxoglutarate, or oxoglutarate [13], which plays essential roles in the living organism: (1) It coordinates carbon and nitrogen balance by incorporating nitrogen into various compounds, thus preventing their accumulation in the body [14]. (2) As a central player in the tricarboxylic acid (TCA) cycle, AKG helps in producing energy-rich ATP molecules [15]. (3) It regulates critical cellular signaling cascade, including the genes AMP-Activated Protein Kinase (AMPK) and the mammalian target of rapamycin (mTOR), which are crucial for cell growth and metabolism pathways [16]. (4) AKG serves as an antioxidant, aiding in the breakdown of fats, amino acids, and glucose [17]. (5) It also acts as a cofactor for the ten-eleven translocase (TET) enzymes, influencing epigenetic changes [18]. (6) AKG influences stem cell differentiation and promotes longevity and fertility in various organisms [18]. Moreover, emerging evidence has shown that exogenous AKG application promotes ammonia utilization, carbon and nitrogen metabolism, and increases crop yield [19,20,21]. In addition, AKG has been reported to enhance plant stress resistance by reducing oxidative stress markers and boosting antioxidant defenses, contributing to better plant growth and adaptability in different challenging environments [22,23,24,25,26,27].

Poplars (*Populus* spp.) serve multiple roles in agriculture and forestry, offering fuelwood, timber, plywood, sports goods, and papermaking raw material [28]. They are also a valuable source of lignocellulosic biomass for biofuel [29]. As an important greening and afforestation tree species, poplar plantations have been developed worldwide. In China, where poplars have been cultivated for over 1500 years, there is an ongoing expansion of its plantation areas. However, with limited available arable land, many fast-growing plantations, including those for poplars, are established on marginal agricultural lands or wastelands [30]. As such areas are increasingly affected by ammonia pollution [31], a significant risk has been posed to the productivity and health of the local plantations, particularly poplar plantations, since they are sensitive to ammonium [32,33].

AKG, as aforementioned, has been applied to various crops to mitigate the harmful effects of ammonia by acting as a ‘scavenger’—a substance that helps neutralize and utilize ammonia efficiently. However, how AKG affects poplar trees when they face high ammonium levels remains unknown. To address this question, we examined several key indicators of plant health, including biomass production, photosynthesis efficiency, and the balance of carbon and nitrogen in poplars planted under excessive ammonium conditions. We also looked at the activity of enzymes associated with these processes and the gene expression patterns that were triggered.

## 2. Results

### 2.1. Effects of AKG on Poplar Growth under High Ammonium Conditions

**Figure 1:**
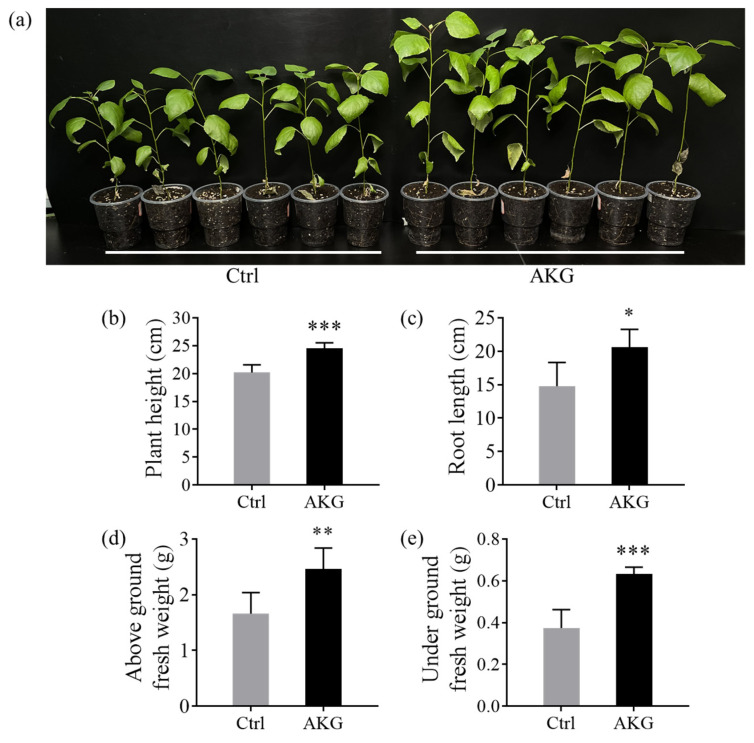
Effects of the exogenous application of AKG on the growth performance of poplar *Nanlin 895* under high ammonium conditions. (**a**) Phenotypic characterization of poplar *Nanlin 895* after two months of treatment, (**b**) plant height (cm plant^−1^), (**c**) root length (cm plant^−1^), (**d**) the above-ground biomass (fresh weight plant^−1^), and (**e**) the underground biomass (fresh weight plant^−1^) of poplar *Nanlin 895*. Poplar plantlets were watered with 5 mM NH_4_Cl, simulating high/excess ammonia exposure. Ctrl: foliar spray of water; AKG: foliar spray of 5 mM α-ketoglutarate. The data were derived from triplicate experiments with standard deviation plotted. Asterisks above error bars denote significant differences between treatments (* *p* < 0.05, ** *p* < 0.01 and *** *p* < 0.001).

To simulate high/excess ammonia exposure, poplar *Nanlin 895* plants were grown under controlled conditions, irrigating with elevated levels of ammonium solution (i.e., 5 mM NH_4_Cl as described in Section 4.1). In this context, a foliar spray of AKG (5 mM) or water, serving as the control (Ctrl) was performed. After a two-month regimen, phenotypic assessments revealed that AKG markedly bolstered the growth of poplar *Nanlin 895* (Figure 1a). Notably, application of AKG resulted in a 21.36% increase in plant height and a 39.32% extension in root length relative to the Ctrl (Figure 1b,c). Furthermore, the fresh weight of the aerial and subterranean tissues of the poplars in the AKG-treated group increased by 48.45% and 68.92%, respectively (Figure 1d,e). These findings underscore the significant enhancement of growth performance in poplar *Nanlin 895* when supplemented with AKG under conditions of high ammonium.

### 2.2. Effects of AKG on Photosynthetic and Chlorophyll Fluorescence Parameters of Poplars Exposed to High Levels of Ammonium

**Figure 2:**
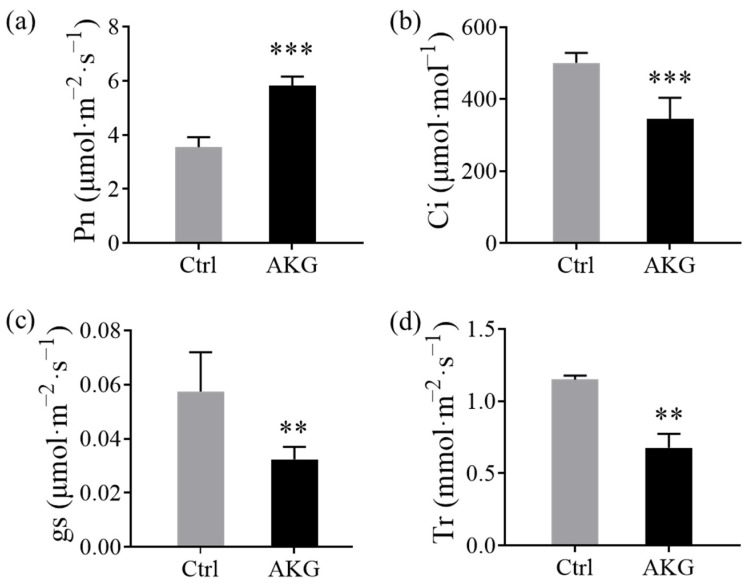
Effects of the exogenous application of AKG on the photosynthetic parameters of poplar *Nanlin 895* under high ammonium conditions. (**a**) Net photosynthetic rate (Pn), (**b**) intercellular CO_2_ concentration (Ci), (**c**) stomatal conductance (gs), and (**d**) transpiration rate (Tr). Poplar plantlets were watered with 5 mM NH_4_Cl, simulating high/excess ammonia exposure. Ctrl: foliar spray of water; AKG: foliar spray of 5 mM α-ketoglutarate. Values are the mean ± SD with three replicates. Asterisks above error bars denote significant differences between treatments (** *p* < 0.01 and *** *p* < 0.001).

The photosynthetic rate is a critical determinant of plant growth vigor. To understand the impact of AKG on poplar growth, we analyzed variations in the net photosynthetic rate (Pn), the intercellular CO_2_ concentration (Ci), the stomatal conductance (gs), and the transpiration rate (Tr). Our data (Figure 2) showed that AKG treatment resulted in a substantial elevation in the Pn (64.11%) compared to the control. Conversely, there was a notable decline in the Ci, gs, and Tr values by 43.55%, 31.20%, and 41.04%, respectively. These results implicate that AKG exerts a pronounced impact on the photosynthetic capacity and CO_2_ assimilation rate in poplars exposed to high ammonium.

**Figure 3:**
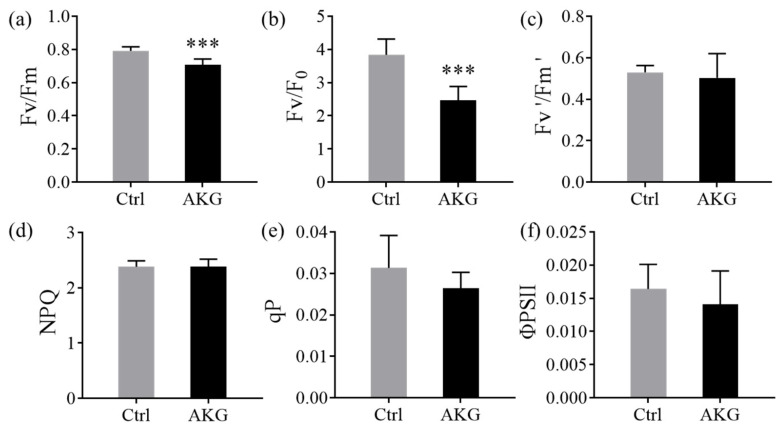
Effects of the exogenous application of AKG on the chlorophyll fluorescence of poplar *Nanlin 895* under high ammonium conditions. (**a**) Maximum PSII quantum yield (F_v_/F_m_), (**b**) PSII activity (F_v_/F_0_), (**c**) effective photochemical efficiency (F_v_’/F_m_’), (**d**) non-photochemical quenching (NPQ), (**e**) photochemical quenching coefficient (qP), and (**f**) photosystem II (PSII). Poplar plantlets were watered with 5 mM NH_4_Cl, simulating high/excess ammonia exposure. Ctrl: foliar spray of water; AKG: foliar spray of 5 mM α-ketoglutarate. Values are the mean ± SD with three replicates. Asterisks above error bars denote significant differences between treatments (*** *p* < 0.001).

Furthermore, we quantified the chlorophyll fluorescence parameters to discern the effects of AKG on the photochemical apparatus of poplar *Nanlin 895*. As shown in Figure 3, the AKG treatment correlated with a decrease of 10.13% in the maximum PSII quantum yield (F_v_/F_m_) and a 35.51% reduction in PSII operating efficiency (F_v_/F_0_), compared with the control. Nonetheless, the effective quantum yield of PSII photochemistry (F_v_’/F_m_’), along with non-photochemical quenching (NPQ), and the photochemical quenching coefficient (qP), exhibited marginal alterations. These observations suggest that the exogenous application of AKG might exert a limited effect on the actual efficiency of light energy conversion, the photosynthetic process, and the photoprotective mechanisms in poplar *Nanlin 895* under high ammonium exposure.

### 2.3. Effects of AKG on Enzymes Related to Carbon and Nitrogen Metabolism in Poplars Exposed to High Ammonium

**Figure 4:**
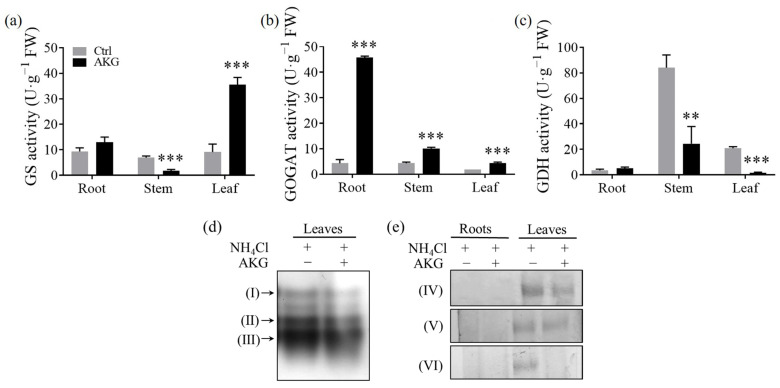
Effects of exogenous AKG on the carbon and nitrogen metabolism enzymes’ activity in the roots, stems, and leaves of poplar *Nanlin 895* exposed to high ammonium. (**a**) Glutamine synthetase (GS) activity, (**b**) glutamate synthase (GOGAT) activity, (**c**) glutamate dehydrogenase (GDH) activity, and (**d**) the on-gel aspartate aminotransferase (AspAT) activity assay. (**e**) Zymogram analysis of the enzymes isocitrate dehydrogenase (ICDH) and NADP-malic enzyme (ME). Poplar plantlets were watered with 5 mM NH_4_Cl, simulating high/excess ammonia exposure. Ctrl: foliar spray of water; AKG: foliar spray of 5 mM α-ketoglutarate; FW: fresh weight. (I) Mitochondrial AspAT isoform. (II) Cytoplasmic AspAT isoform. (III) Chloroplast AspAT isoform. (IV) NADP-ICDH activity. (V) NAD-ICDH activity. (VI) NADP-ME activity. The experiment was repeated thrice, with similar results. One representative activity gel was presented. Values are the mean ± SD with three replicates. Asterisks above error bars denote significant differences between treatments (** *p* < 0.01 and *** *p* < 0.001).

The glutamine synthetase (GS)/glutamate synthase (GOGAT) and the glutamate dehydrogenase (GDH)/aspartate aminotransferase (AspAT) are two pathways of ammonium assimilation that facilitate the conversion of AKG and ammonium into glutamate and glutamine, thereby mitigating the stress induced by high ammonium levels [14]. Our study unveiled a significant variation (Figure 4) in GS enzyme activity that was tissue-specific in poplar *Nanlin 895* following AKG treatment, as opposed to the Ctrl. Notably, AKG exposure led to an over two-fold increment of GS activity in the foliage. In contrast, it resulted in a 74.21% reduction in the stems, with the root activity remaining unaffected (Figure 4a). In contrast, GOGAT activity exhibited an increase across all tissue types, with the most pronounced enhancement occurring in the roots at a nine-fold increase, followed by the stems and leaves, which doubled and increased by one and a half times compared with the Ctrl (Figure 4b); inversely, GDH activity experienced a decline of 70.92% in the stems and a marked decrease (92.50%) in the leaves, yet it did not alter in the roots in comparison with the Ctrl (Figure 4c).

Assessment of AspAT activity via the on-gel enzyme assays revealed that AKG treatment attenuated the activity of this enzyme, including its cytosolic, plastidic, and mitochondrial isoforms, within the leaf tissue (Figure 4d and Figure S3). Moreover, zymogram analysis for the key enzymes, NADP-dependent isocitrate dehydrogenase (NADP-ICDH), NAD-dependent isocitrate dehydrogenase (NAD-ICDH), and NADP-dependent malic enzyme (NADP-ME) associated with the TCA cycle, demonstrated that AKG administration suppressed the activity of these enzymes in the leaves. In contrast, their activity in the roots was nearly non-detectable, irrespective of AKG treatment (Figure 4e and Figure S3). Given that these enzymes are instrumental in the generation of AKG via the TCA cycle, their reduced activity indicates that exogenous AKG supplementation may take the bypass of TCA cycle anaplerosis to avoid metabolites and/or energy waste in plants subjected to high ammonium stress.

### 2.4. Effects of AKG on the Inorganic Nitrogen Content in Poplars under High Ammonium Conditions

**Figure 5:**
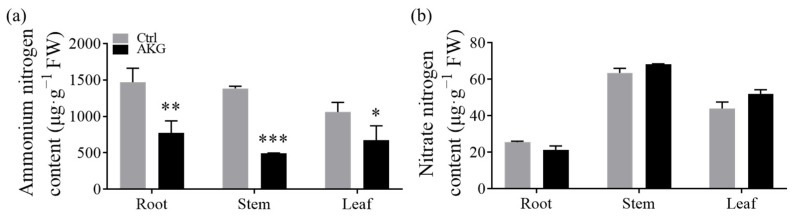
Effects of exogenous AKG on the content of inorganic nitrogen in the roots, stems, and leaves of poplar *Nanlin 895* under high ammonium. (**a**) Ammonium–nitrogen content. (**b**) Nitrate–nitrogen content. Poplar plantlets were watered with 5 mM NH_4_Cl, simulating high/excess ammonia exposure. Ctrl: foliar spray of water; AKG: foliar spray of 5 mM α-ketoglutarate; FW: fresh weight. Values are the mean ± SD with three replicates. Asterisks above the error bars denote a significant difference between the treatments (* *p* < 0.05, ** *p* < 0.01 and *** *p* < 0.001).

Plants are physiologically incapable of sustaining elevated intracellular ammonium concentrations; thus, they must swiftly assimilate the absorbed or synthesized ammonium into alternative nitrogenous compounds to mitigate ammonium toxicity [34]. In our investigation into the role of AKG in the detoxification of ammonium, we quantified the levels of nitrate and ammonium nitrogen in poplar *Nanlin 895* that were treated with AKG in the presence of high ammonium. As revealed in Figure 5a, exogenously applying AKG led to a marked reduction in ammonium nitrogen content, demonstrating decreases of 47.31% in the roots, 64.41% in the stems, and 36.52% in the leaves upon high ammonium treatment. Conversely, the nitrate nitrogen levels remained largely unaffected by AKG treatment compared with the Ctrl (Figure 5b).

### 2.5. Impacts of AKG on Nitrogenous Compounds and Carbohydrates in Poplars with High Ammonium Exposure

**Figure 6:**
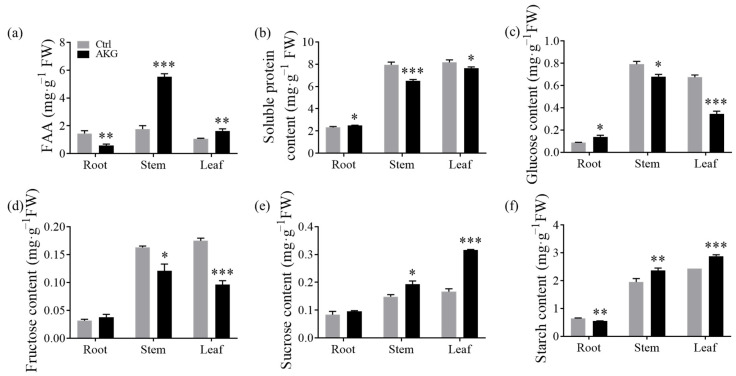
Effects of exogenous AKG on nitrogen and carbon metabolites in poplar *Nanlin 895* with high ammonium exposure. (**a**) Free amino acid content, (**b**) soluble protein content, (**c**) glucose content, (**d**) fructose content, (**e**) sucrose content, and (**f**) starch content. Poplar plantlets were watered with 5 mM NH_4_Cl, simulating high/excess ammonia exposure. Ctrl: foliar spray of water; AKG: foliar spray of 5 mM α-ketoglutarate; FW: fresh weight. Values are the mean ± SD with three replicates. Asterisks above error bars denote significant differences between treatments (* *p* < 0.05, ** *p* < 0.01 and *** *p* < 0.001).

AKG plays a critical function in the intersection of carbon and nitrogen metabolic pathways [14]. Analysis of free amino acid levels in poplar *Nanlin 895* revealed that AKG treatment caused a substantial decline (by 59.33%) of total amino acid content in the root tissues. Conversely, there was a pronounced increase in total amino acids content in the stems (213.42%) and leaves (53.56%) when compared to the Ctrl (Figure 6a). Meanwhile, the soluble protein content slightly increased in roots (4.21%) but decreased in both stems (16.82%) and leaves (5.93%) in the AGK-treated plants compared with the Ctrl (Figure 6b). In terms of carbon compounds, the concentrations of fructose and sucrose did not significantly change in the roots of poplar *Nanlin 895* applied with AKG. In contrast, glucose content was elevated by 60.75%, and starch decreased by 14.54% compared with the Ctrl (Figure 6c–f). In the stem tissues, both sucrose and starch concentrations were increased by 30.34% and 21.01%, respectively, with glucose and fructose levels diminishing by 14.17% and 25.83% in response to AKG treatment. In the leaf tissues, both sucrose and starch contents saw substantial increases of 89.89% and 18.26%, respectively, whereas glucose and fructose concentrations declined sharply by 48.75% and 44.96%, respectively, under AKG treatment compared with the Ctrl.

### 2.6. Effects of AKG on Energy Budget in Poplars with High Ammonium Exposure

**Figure 7:**
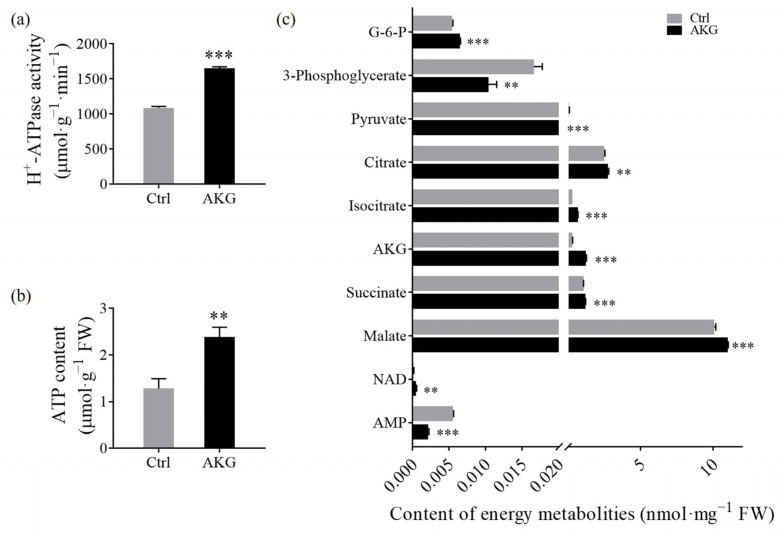
Effects of exogenous AKG on energy budget in the leaves of poplar *Nanlin 895* under high ammonium conditions. (**a**) H^+^-ATPase activity. (**b**) ATP content. (**c**) The abundance of relevant energy metabolites. Poplar plantlets were watered with 5 mM NH_4_Cl, simulating high/excess ammonia exposure. Ctrl: foliar spray of water; AKG: foliar spray of 5 mM α-ketoglutarate; FW: fresh weight; G-6-P: glucose-6-phosphate; NAD: nicotinamide adenine dinucleotide; AMP: adenosine monophosphate. Values are the mean ± SD with three replicates. Asterisks above error bars denote significant differences between treatments (** *p* < 0.01 and *** *p* < 0.001).

To elucidate the influence of AKG on energy homeostasis within poplar *Nanlin 895*, we assessed the enzyme activity of H^+^-ATPase alongside the concentrations of key energy metabolites. The analysis revealed that AKG application resulted in a remarkable increase in both H^+^-ATPase activity (Figure 7a) and ATP levels (Figure 7b) compared to the Ctrl, suggesting an AKG-mediated enhancement of energy production in conditions of high ammonium. Further metabolic quantification analysis using LC-MS indicated significant alterations in the levels of several energy metabolites. Specifically, in AKG-treated plants, there was an increase of 19.29% in glucose-6-phosphate (G-6-P), 10.05% in citrate, 124.06% in isocitrate, 326.38% in AKG itself, 13.40% in succinate, 9.27% in malate, and 244.78% in nicotinamide adenine dinucleotide (NAD), relative to the Ctrl. Conversely, there was a reduction in the levels of 3-phosphoglycerate, pyruvate, and adenosine monophosphate (AMP) by 37.19%, 45%, and 61.67%, respectively (Figure 7c). These findings indicate that AKG exerted a significant modulatory effect on glycolysis, the TCA cycle, and the respiratory pathway’s energy metabolism.

### 2.7. Effects of AKG on the Enzymatic Antioxidant Capacity of Poplars under High Ammonium Conditions

**Figure 8:**
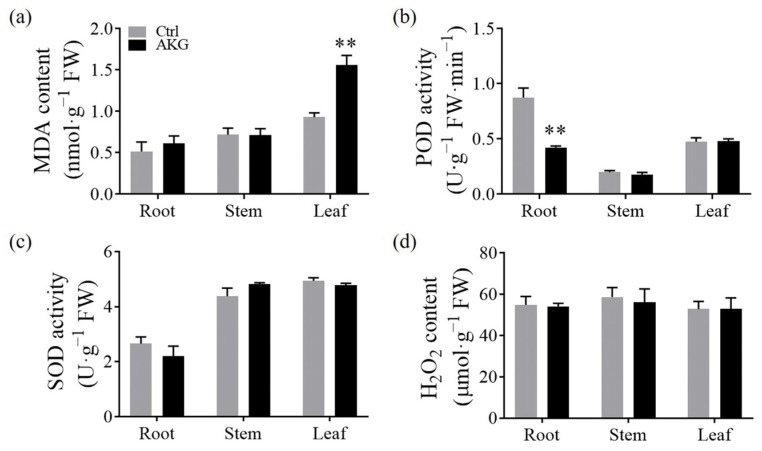
Impact of exogenous AKG on the enzymatic antioxidant capacity of poplar *Nanlin 895* under high ammonium. (**a**) MDA content, (**b**) POD activity, (**c**) SOD activity, and (**d**) H_2_O_2_ content. Poplar plantlets were watered with 5 mM NH_4_Cl, simulating high/excess ammonia exposure. Ctrl: foliar spray of water; AKG: foliar spray of 5 mM α-ketoglutarate; FW: fresh weight; MDA: malondialdehyde; POD: peroxidase; SOD: superoxide dismutase; H_2_O_2_: hydrogen peroxide. Values are the mean ± SD with three replicates. Asterisks above error bars denote significant differences between treatments (** *p* < 0.01).

To ascertain the impact of AKG on the redox balance, we quantified the levels of oxidative stress indicators, including hydrogen peroxide (H_2_O_2_) and malondialdehyde (MDA), and detected the activities of the classical antioxidant enzymes peroxidase (POD) and superoxide dismutase (SOD) in poplar *Nanlin 895*. The investigation revealed that, following AKG treatment, there was a 67.74% increase in MDA levels exclusively in the leaf tissues, with the roots and stems showing no significant alterations compared to the Ctrl (Figure 8a). In contrast, POD activity experienced a 51.61% decrease in the roots, while it exhibited no appreciable change in the stems and leaves relative to the Ctrl (Figure 8b). The activities of SOD (Figure 8c) and the concentration of H_2_O_2_ (Figure 8d) remained constant across all tissues assessed. These findings suggest that the addition of exogenous AKG exerts a minimal effect on the classical enzymatic antioxidant defenses in poplar *Nanlin 895* under conditions of high ammonium stress.

### 2.8. Effects of AKG on the Carbon and Nitrogen Metabolism-Related Genes in Poplars with High Ammonium Exposure

**Figure 9:**
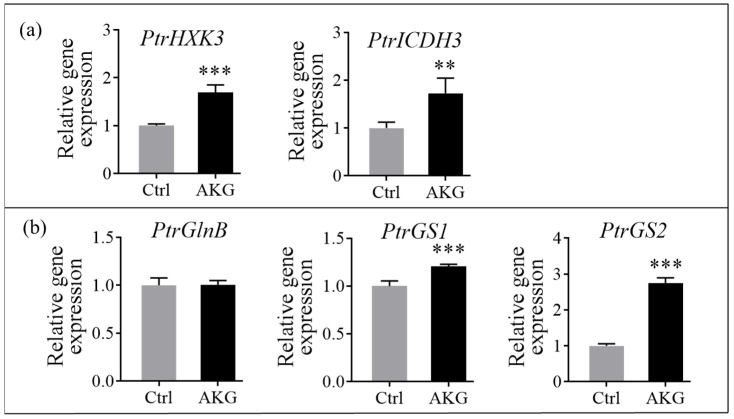
Relative expression of carbon and nitrogen metabolism-related genes in response to AKG treatment in the leaves of poplar *Nanlin 895* under high ammonium. (**a**) Key carbon flux regulation genes *PtrHXK3* and *PtrICDH3*. (**b**) The nitrogen metabolism-related genes *PtrGlnB*, *PtrGS1*, and *PtrGS2*. Poplar plantlets were watered with 5 mM NH_4_Cl, simulating high/excess ammonia exposure. Ctrl: foliar spray of water; AKG: foliar spray of 5 mM α-ketoglutarate. *Ptr*: *Populus trichocarpa*; *HXK*: *hexokinase*; *ICDH*: *isocitrate dehydrogenase*; *GlnB*: *nitrogen regulatory protein PII*; *GS*: *glutamine synthetase*. Populus *UBIC* was employed as an internal control for RT-qPCR analysis. The data are presented as the mean ± SD with three biological replicates. Asterisks above error bars denote significant differences between treatments (** *p* < 0.01, and *** *p* < 0.001).

To identify the genes responsible for ammonia detoxification influenced by AKG treatment at the cellular metabolism level, we quantified the relative mRNA expression levels of pivotal genes involved in the carbon and nitrogen metabolism pathways by quantitative real-time PCR (qPCR) in poplar *Nanlin 895*, both with and without AKG treatment. Under conditions of high ammonium stress, AKG treatment resulted in a significant upregulation in critical genes governing carbon flux, with hexokinase (*PtrHXK3*) and isocitrate dehydrogenase (*PtrICDH3*) transcripts increasing by 69.76% and 72.82%, respectively, in the leaves compared to the Ctrl (Figure 9a). Additionally, the expression of genes *PtrGS1* and *PtrGS2*, integral to nitrogen assimilation, were enhanced by 20.49% and 174.21%, respectively, in the leaves (Figure 9b). In contrast, the transcript levels of the AKG sensor gene, *nitrogen regulatory protein PII* (*PtrGlnB*, named P_II_), exhibited negligible responsiveness to AKG treatment when compared with the Ctrl (Figure 9b).

### 2.9. Effects of AKG on Energy Metabolism-Related Genes in Poplars with High Ammonium Exposure

**Figure 10:**
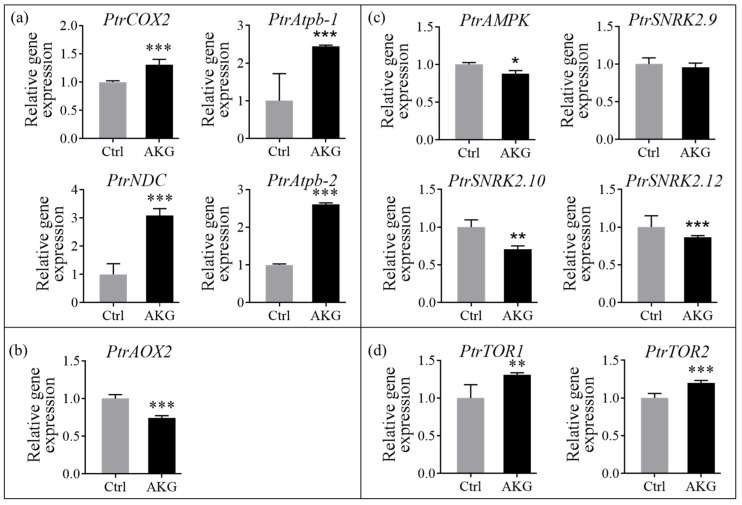
Relative expression of genes associated with energy metabolism in response to AKG in the leaves of poplar *Nanlin 895* under high ammonium. (**a**) ATP synthesis genes. (**b**) ATP dissipation gene. (**c**) ATP signaling genes. (**d**) Growth regulatory genes. Poplar plantlets were watered with 5 mM NH_4_Cl, simulating high/excess ammonia exposure. Ctrl: foliar spray of water; AKG: foliar spray of 5 mM α-ketoglutarate. *Ptr*: *Populus trichocarpa*; *COX*: *cytochrome c oxidase*; *Atpb*: *β subunit of ATP synthase*; *NDC*: *NADH ubiquinone oxidoreductase*; *AOX*: *alternative oxidase*; *AMPK*: *adenosine monophosphate-activated protein kinase*; *SNRK*: *sucrose nonfermenting 1-related kinase*; *TOR*: *target of rapamycin*. Populus *UBIC* was employed as an internal control for RT-qPCR analysis. The data are presented as the mean ± SD with three replicates. Asterisks above error bars denote significant differences between the treatments (* *p* < 0.05, ** *p* < 0.01, and *** *p* < 0.001).

The expression level of ATP synthesis-related genes encoding cytochrome c oxidase (COX), the β subunit of ATP synthase (Atpb), and NADH ubiquinone oxidoreductase (NDC) [16,35] were detected in the leaves of poplar *Nanlin 895* treated with or without AKG under high ammonium stress. The qPCR analyses indicated significant modulation of these genes (Figure 10). Specifically, there was an upregulation in *PtrCOX2*, *PtrAtpb-1* and *PtrAtpb-2*, and *PtrNDC*, with increases in transcript levels of 30.90%, 144.31%, 161.07%, and 208.49%, respectively, in the AKG treated plants compared with the Ctrl (Figure 10a). Conversely, the expression of the gene encoding alternative oxidase (AOX) associated with ATP dissipation [36], i.e., *PtrAOX2*, was reduced by 25.81% (Figure 10b). Similarly, the transcripts of adenosine monophosphate-activated protein kinase (AMPK) and sucrose nonfermenting 1-related kinase (SNRK), key genes involved in energy sensing [16], i.e., the *PtrAMPK*, *PtrSNRK2.10*, and *PtrSNRK2.12*, were downregulated by 12.31%, 29.03%, and 13.68%, respectively, while the transcript level of *PtrSNRK2.9* was not significantly altered by AKG treatment (Figure 10c). In addition, AKG treatment stimulated the transcription of the growth-regulatory genes encoding target of rapamycin (*TOR*) [16], with *PtrTOR1* and *PtrTOR2* showing elevations of 30.70% and 19.81%, respectively (Figure 10d).

## 3. Discussion

### 3.1. Application of AKG Promotes Ammonium Assimilation in Poplars with High Ammonium Exposure

AKG is an essential cellular metabolite participating in various biological functions, including nitrogen translocation, substrate and redox metabolism, amino acid biosynthesis, and gene expression modulation [13,37,38]. The regulatory capacity of AKG over nitrogen and carbon metabolism has been well-documented in *Escherichia coli* and cyanobacteria [14,39]. However, its role in ameliorating ammonium toxicity in woody plants, such as poplars, remains unexplored. Our findings suggest that AKG supplementation significantly enhances growth parameters in poplar *Nanlin 895* under high ammonium levels, evidenced by notable increases in height and biomass (Figure 1). These observations were in alignment with prior reports that demonstrate the growth-promoting effects of exogenous AKG application in various crops [19,20,22,23,24,25,26,27], implying that the role of AKG in ammonium detoxification is conserved in different organisms.

Plants utilize two primary pathways for ammonium assimilation: the GS/GOGAT cycle and the GDH-mediated reaction [14,38]. Our investigation showed that AKG administration led to a decrease in GDH and AspAT activities in the leaves (Figure 4c,d), akin to findings in tomato plants [23], indicating that the GDH-mediated reaction is a low ammonium affinity pathway [40] in poplars treated with AKG under high ammonium stress. By contrast, exogenous AKG enhanced the expression of *PtrGS1* and *PtrGS2* (Figure 9b) and stimulated GS/GOGAT enzyme activities in the leaves (Figure 4a,b), suggesting that the GS/GOGAT cycle is preferential for AKG-mediated ammonium detoxification in poplars. While AKG did not significantly alter the transcript levels of the nitrogen regulatory protein P_II_ (*GlnB*) (Figure 9b), which is known to sense AKG and play essential roles in modulating GS enzyme activity [38,41], it raises the possibility that AKG’s regulatory influence on the GS/GOGAT cycle in poplars might occur independently of the AKG/P_II_ signaling pathway. Nevertheless, the potential for AKG to exert post-transcriptional or post-translational regulation on P_II_, as demonstrated in other systems [42], cannot be excluded.

In addition, an increase in free amino acid levels (Figure 6a) and a reduction in ammonium content (Figure 5a) were observed, suggesting AKG’s significant role in ammonia assimilation. Notably, the considerable increase in free amino acid content in stems could act as a detoxifying mechanism [38] and/or indicate increased amino acid transport between root and shoot.

### 3.2. AKG Promotes Carbon Metabolism in Response to High Ammonium Exposure

AKG occupies a central position in the TCA cycle, a crucial pathway in cellular carbon metabolism. As the main carbon backbone for nitrogen assimilation [38], AKG plays a pivotal role in balancing catabolic and anabolic processes, thereby regulating photosynthesis, carbon metabolism, and plant growth [43]. Prior research has evidenced that exogenous AKG can enhance photosynthesis [44] and carbohydrate production [45]. Consistent with these findings, our study observed an increase in the photosynthetic parameter Pn upon AKG supplementation (Figure 2), an upregulated expression of the glucose metabolism gene *PtrHXK3* (Figure 9a), and elevated levels of starch, sucrose (Figure 6e,f), and glucose-6-phosphate (Figure 7b), accompanied by altered glucose and fructose consumption in poplars under high ammonium stress (Figure 6c,d). Additionally, AKG treatment led to an accumulation of TCA cycle intermediates, including citrate, isocitrate, succinate, and malate (Figure 7c), supporting AKG’s role in carbon metabolism regulation under ammonium-rich conditions.

The synthesis of organic nitrogen compounds from ammonium necessitates AKG, typically generated by NAD^+^ or NADP^+^-dependent ICDH [46]. Although the *PtrICDH3* gene expression was enhanced (Figure 9a), a paradoxical decrease in ICDH enzyme activity was noted (Figure 4e), suggesting that the exogenous provision of AKG might circumvent the need for endogenous AKG production via ICDH [46]. Given the TCA cycle’s role in supplying precursors for biosynthesis [47], it can be inferred that exogenous AKG may compensate for the diversion of TCA intermediates towards biosynthetic pathways under ammonium surplus, facilitating sustained TCA cycle function without reliance on in vivo AKG biosynthesis.

Furthermore, AKG application under high ammonium stress was found to inhibit NADP-ME enzyme activity (Figure 4e), and decrease the levels of glycolytic intermediates, such as 3-phosphoglycerate and pyruvate (Figure 7c). Since NADP-ME is responsible for the conversion of malate to pyruvate [48], the observed reductions suggest a restricted carbon flux into the glycolytic pathway following AKG treatment. These observations imply that AKG addition under ammonia stress preferentially bolsters the TCA cycle over glycolysis, a strategy that may be more efficacious for metabolism and energy production [43,49]. Such an approach could allow for immediate adjustments in TCA flux, potentially facilitating an improved growth response in poplars to high ammonium levels without significant enzymatic alterations.

### 3.3. Promotion of Energy Metabolism by AKG in Poplars under High Ammonium Conditions

ATP is the primary energy source for many biochemical reactions, such as protein synthesis, metabolic process, RNA synthesis, and cell volume increase [50]. Because ATP is dynamically synthesized and consumed in considerable amounts [51], stable ATP levels (indicative of robust ATP homeostasis) are crucial for optimal growth and defense mechanisms [50,52]. In this study, it was found that AKG supplementation resulted in upregulated transcript levels of ATP synthesis genes [16,35,53], *PtrCOX2*, *PtrNDC*, *PtrAtpb-1*, and *PtrAtpb-2* (Figure 10a), indicating that ATP biosynthesis was enhanced in the leaves of poplar *Nanlin 895* exposed to high ammonium. Conversely, transcripts of the ATP catabolism gene [36] *PtrAOX2* were downregulated (Figure 10b). Concurrently, a significant increase in H^+^-ATPase activity and ATP content was detected (Figure 7a,b). These findings suggest that AKG supplementation is conducive to sustaining optimal ATP levels by promoting ATP synthesis and curtailing ATP degradation under high ammonium conditions. In addition, the induction of the master growth regulators *PtrTOR1* and *PtrTOR2* and the suppression of stress-responsive gene *SnRK* by AKG (Figure 10c,d), implicate that the TOR/SnRK signaling axis is involved in modulating the balance between growth and stress response conditioned by energetic and nutritional status [54].

Furthermore, a reduction in soluble protein levels following AKG treatment was observed (Figure 6b), indicating a strategic reduction in biosynthetic ATP expenditure [55]. This aligns with the notion that the cellular economy is managed by producing proteins essential for immediate physiological needs, such as detoxifying excess ammonium [56]. However, it cannot be excluded that the lower protein content might be due to a “dilution effect” of the biomass since the higher biomass production after spraying AKG will also demand higher energy in the form of ATP. Our results suggest that AKG administration under high ammonium conditions may enhance energy availability, facilitating efficient utilization of carbon molecules for ammonia detoxification and supporting cellular metabolic demands.

### 3.4. Improvement of the Non-Enzymatic Antioxidant Capacity by AKG under High Ammonium Conditions

AKG has been implicated in mitigating oxidative stress in several model organisms. In animal systems, AKG facilitates ammonia detoxification by attenuating oxidative stress markers [57] and enhancing the concentrations of antioxidative enzymes [25]. Contrasting with these findings, our study in poplar *Nanlin 895*, reveals that AKG supplementation insignificantly altered the activity of the enzymatic antioxidants (Figure 8), suggesting a divergent mechanism of AKG-mediated ammonia detoxification in poplars. The observed increment in MDA content in the leaves under high ammonium conditions (Figure 8a) might potentially be linked to an augment in reactive oxygen species (ROS) associated with enhanced ATP synthesis and respiratory chain activity, particularly at complexes I and III [58], as indicated by the elevated transcript levels of *PtrCOX2* and *PtrNDC* (Figure 10a).

Moreover, AKG treatment resulted in a notable increase in TCA cycle intermediates such as citrate, succinate, malate, and endogenous AKG (Figure 7c), compounds recognized for their antioxidative properties and role in oxidative stress regulation [59]. The unchanged H_2_O_2_ content upon AKG application (Figure 8d) suggests an efficient utilization of AKG in the detoxification reaction with H_2_O_2_ to yield succinate and water within the TCA cycle, thereby enhancing the substrate availability for complex II (succinate dehydrogenase) to promote energy production [37]. Our results indicate that the elevation of TCA cycle intermediates under AKG supplementation may not only support respiratory metabolism but could also augment the non-enzymatic antioxidant capacity [25,57,60]. Collectively, it underscores the multifaceted role of AKG in enhancing the antioxidant defenses of poplar *Nanlin 895* when subjected to high ammonium stress, potentially through a mechanism distinct from those observed in animals and humans [25].

## 4. Materials and Methods

### 4.1. Plant Growth Conditions and Treatment

In the current study, we investigated the functions of ammonium and AKG on the growth of poplar *Nanlin 895* (*Populus deltoides* × *Populus euramericana* cv. ‘*Nanlin 895*’) planted in a soil matrix mix of peat moss, vermiculite, and perlite (3:1:1, by volume) in 250 mL pots. Unless otherwise indicated, the seedlings of poplar *Nanlin 895* were rooted on the half strength MS medium for five weeks. The plants that rooted were moved to a water tank to eliminate the internal N for one week. Then, plants with similar heights were selected for the following experiments in a climate chamber (RDN-1000, Ningbo Dongnan Instrument Co., Ltd., Ningbo, China) with a period of 16 h light to 8 h dark, at 25 °C/20 °C. Before the treatment, a preliminary hydroponic experiment [61] was initiated to determine the appropriate concentrations (1, 3, 5, or 10 mM) of ammonium chloride (NH_4_Cl) for treatment. Poplar *Nanlin* 895 showed impaired growth at 5 mM NH_4_Cl and severe disturbance at 10 mM NH_4_Cl (Figure S1). Aligning with the literature that classifies concentrations above 3 mM of NH_4_^+^-N as excessive for plants [28,62], we therefore chose 5 mM NH_4_Cl for further investigation. For AKG treatments, we sprayed various concentrations (0, 0.05, 0.5, 5, and 10 mM) of AKG to each plant and observed optimal growth at 5 mM AKG (Figure S2). Resultantly, both 5 mM AKG foliar spray (approximately 5 mL plant^−1^) and 5 mM NH_4_Cl watering (20 mL pot^−1^) were applied triweekly to the poplars. Control plants (Ctrl) received only water sprays. At least six plants were employed for each treatment, and the treatment lasted for two months. After that, uniformly grown plants were selected for harvesting. The collected samples (i.e., roots, stems, and leaves) were immediately frozen in liquid nitrogen, and stored at −80 °C for subsequent analysis. The whole experiment was replicated three times.

### 4.2. Measurement of Chlorophyll Fluorescence and Photosynthetic Parameters

Net photosynthetic rate (Pn, μmol CO_2_·m^−2^·s^−1^) along with the intercellular carbon dioxide concentration (Ci, μmol CO_2_·mol^−1^), the stomatal conductance (gs, μmol·m^−2^·s^−1^), and the transpiration rate (Tr, mmol H_2_O·m^−2^·s^−1^) of poplar *Nanlin 895* seedlings were detected by the usage of a portable photosynthesis system, the Li-6400 (LI-COR Inc., Lincoln, NE, USA), according to the user manual. The third fully extended leaf was chosen to measure the chlorophyll fluorescence by using a MINI-PAM-II ultra-portable modulated chlorophyll fluorescence analyzer (Zequan Technology Co., Ltd., Shanghai, China), following the instructions of the user manual. Fluorescence parameters were calculated as described previously [63].

### 4.3. Determination of Enzyme Activities

Glutamine synthetase (GS), glutamate synthase (GOGAT), glutamate dehydrogenase (GDH), and aspartate aminotransferase (AspAT) activities were assayed in poplar leaves, stems and roots based on the previously described method [61]. In brief, the extraction buffer consists of 10 mM Tris-HCl (pH 7.6), 1 mM EDTA, and 1 mM MgCl_2_, and 1 mM β-mercaptoethanol was added to the tissue samples (approximated 100 mg), which were homogenized and then centrifuged at 12,000 rpm at 4 °C for 20 min. After that, the supernatant was removed and used to determine the enzyme activities. One unit of the GS activity was identified as the formation of 1 µmol of γ-glutamyl hydroxamate per minute. One unit of the GOGAT and GDH were converted to oxidize 1 nmol min^−1^ of NADH. AspAT isoenzyme activities were assayed using Native-PAGE as previously described [64]. The activities of NADP-dependent isocitrate dehydrogenase (NADP-ICDH), NAD-dependent isocitrate dehydrogenase (NAD-ICDH), and NADP-dependent malic enzyme (NADP-ME) were measured based on the BN-PAGE method described previously [65]. The ATPase activity was measured following the protocol in a previous study [66].

### 4.4. Nitrogen Compounds, Carbohydrates and ATP Content Measurements

The nitrate–nitrogen content in poplar roots, stems, and leaves was measured by using the modified Patterson method [67]. In brief, sterile water (1 mL) was added to the tissue sample and heated at 100 °C for 30 min. Then, the supernatant (0.1 mL) was transferred to a clean tube (10 mL) containing 5% sulfuric acid (0.4 mL); after that, the reaction was cooled down to the ambient temperature and 9.5 mL of 8% NaOH was added. The absorbance of the solution was recorded spectrophotometrically at 410 nm. The ammonium was extracted by the extraction buffer containing 100 mM HCl (1 mL) and chloroform (500 µL), and its content was measured in a spectrophotometer at 620 nm according to the colorimetric method previously described [68]. Protein contents were quantified by the Detergent Compatible Bradford Protein Assay Kit (Beyotime, Shanghai, China) according to the instructions of the user manual, using bovine serum albumin (BSA) as a standard. The absorbance of the solution was recorded in a spectrophotometer at 595 nm wavelength (752Pro, Lengguang, Shanghai, China).

Carbohydrates and the total free amino acid content were determined according to the procedure outlined in an earlier study [69]. In brief, a volume of 1 mL of 80% (*v*/*v*) ethanol was added to the dried sample powder (40 mg), heated at 80 °C, and then the supernatant was collected. This procedure was repeated thrice. The free amino acids were measured with ninhydrin, and the results were expressed in mg gram^−1^ of fresh weight (FW). The sucrose, glucose, fructose, and starch content were determined by the anthrone method [70], and their concentrations were expressed in mg gram^−1^ of fresh weight (FW). The ATP levels were detected using the ATP Content Detection Kit (G0857W, Grace Biotechnology, Suzhou, China), based on the instructions in the user manual.

### 4.5. Determination of the Content of Energy Metabolites

Metabolites were extracted from fresh leaf tissues (200 mg) in 1 mL of pre-cooled methanol-to-acetonitrile-to-water (2:2:1, by volume) by ultrasonication in an ice bath for 30 min. The extraction process was repeated two times, and the solution was combined and incubated at −20 °C for one hour to precipitate proteins; after that, the solution was centrifuged at 12,000 rpm at 4 °C for 20 min. The supernatant was filtered and then aspirated by vacuum drying. For mass spectrometry measurement, adding 200 μL of acetonitrile–aqueous solution (1:1, by volume) for resolution. A mixture of stable isotopically labeled chemicals was utilized as an internal standard.

Chromatographic separation was implemented on a Waters BEH Amide column (100 mm × 2.1 mm, 1.8 μm) using an ultrahigh-performance liquid chromatography (UPLC) system (Vanquish, Thermo, Waltham, MA, USA) that is coupled with a high-resolution mass spectrometer (Q Exactive, Thermo, USA). The mobile phase was composed of 10 mM ammonium acetate solution (A) and acetonitrile (B), at a flow rate of 0.3 mL min^−1^ and a column temperature of 40 °C. The injection volume was 2 μL and the elution gradient (0–9 min) involved a linear change in liquid B from 90% to 40%; At 9–9.1 min, liquid B linearly increased from 40% to 90%. At 9.1–12 min, liquid B was kept at 90%. The samples were placed in an autosampler (4 °C) and analyzed in a random order to mitigate signal fluctuations, with quality control (QC) samples interspersed to ensure system stability and data reliability.

Detection was carried out using the QExactive high-resolution mass spectrometry detection system (Thermo Company), with the following electrospray ionization (ESI) conditions: sheath gas at 40 arbitrary units (arb), auxiliary gas at 10 arb, ion transfer tube temperature at 320 °C, capillary temperature at 350 °C, and ion spray voltage at −2800 V. The full scan-DDMS2 (negative ion) scanning mode was employed. The primary scan range was *m*/*z* 70 to 1000. Metabolite identification was based on the exact mass and retention time matched against the internal metabolite library.

### 4.6. Analysis of Malondialdehyde (MDA), H_2_O_2_ Content, and the Antioxidant Enzyme Activities

The extraction and activities of the antioxidant enzymes peroxidase (POD) and superoxide dismutase (SOD) were determined according to the method described in a previous publication [60]. The H_2_O_2_ content was calculated using titanium following the method described previously [71]. The content of MDA was determined according to an earlier study [60].

### 4.7. Total RNA Extraction and the Quantitative Real-Time PCR (qRT-PCR) Analysis

The total RNA of poplar leaves was extracted from leaf tissues using the Plant RNA Extraction Kit V 1.6 (BIOFIT, Chengdu, China), and the obtained RNA samples were then reverse transcribed into cDNA by the FastKing gDNA Dispelling RT SuperMix (TIANGEN, Beijing, China). Quantitative PCR (qPCR) analysis was conducted using the Applied Biosystems RT-PCR system (Applied Biosystems, Foster City, CA, USA). PCR conditions were as follows: denaturation at 95 °C for 5 s, annealing at 60 °C for 30 s, and extension at 72 °C for 30 s, running with 40 cycles. The reference gene (ubiquitin, XM_002309363. 2) was used to normalize the transcript levels among samples, and the 2^−ΔΔCt^ method was employed to calculate the relative mRNA expression levels [72]. Gene-specific primers were designed by using the Primer 3 program (https://bioinfo.ut.ee/primer3-0.4.0/, accessed on 20 March 2023), and amplification product length was controlled at 100–250 bp; GC content was ranging from 40% to 60%, and the primer Tm value was set at 58–62 °C. Information on the primers used for this study is presented in Table S1.

### 4.8. Statistical Analysis

Microsoft Excel 2016 was utilized for preliminary summary and collection of the original data, and IBM SPSS version 21 analysis software (IBM, Chicago, IL, USA) was applied for data comparison. Independent sample *t*-test was used to estimate the significance level of differences between groups. The charts were drawn using GraphPad Prism 7 (Microsoft, Redmond, DC, USA).

## 5. Conclusions

**Figure 11:**
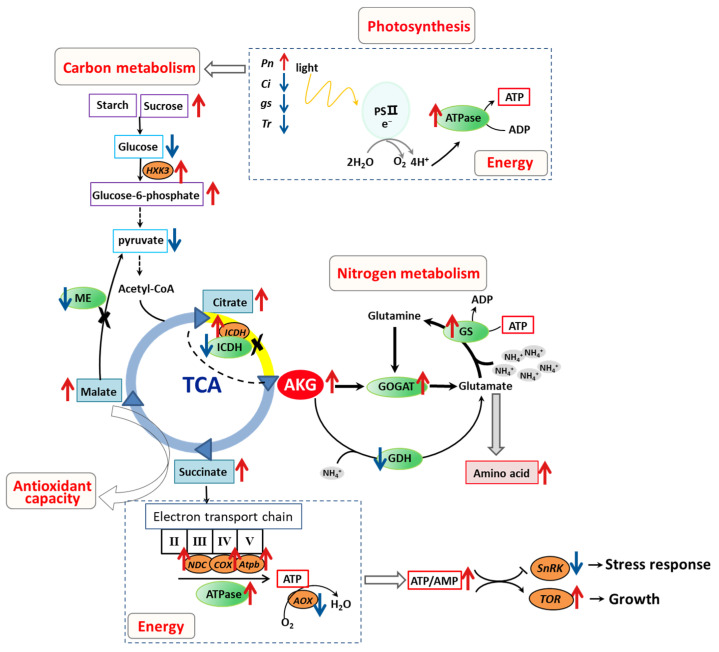
Schematic diagram illustrating possible pathways regulated by AKG in poplar *Nanlin 895* exposed to high ammonium. The model was developed based on the data from the leaves. Red arrows represent elevated metabolites and genes, and blue arrows represent declined metabolites and genes in plants treated with AKG compared to plants treated without AKG. Dashed arrows represent multiple processes; Cross indicate that the step was hindered. GS: glutamine synthetase; GOGAT: glutamate synthase; GDH: glutamate dehydrogenase; ICDH: isocitrate dehydrogenase; *HXK3*: *hexokinase 3*; *COX*: *cytochrome c oxidase*; *Atpb*: *β subunit of ATP synthase*; *NDC*: *NADH ubiquinone oxidoreductase*; *AOX*: *alternative oxidase*; *AMPK*: *adenosine monophosphate-activated protein kinase*; *SNRK*: *sucrose nonfermenting 1-related kinase*; *TOR*: *target of rapamycin*.

The ameliorative effects of AKG on ammonium toxicity in poplar *Nanlin 895* were explored through foliar application. Our results unequivocally indicate that AKG supplementation confers substantial growth benefits, as evidenced by increased stature and biomass accumulation in both the above-ground and underground components of the plants subject to high ammonium exposure. Our data suggest that AKG facilitates a multifaceted detoxification strategy, which is characterized by harmonizing carbon and nitrogen assimilation processes to prevent an overload of ammonia–nitrogen, bolstering metabolic pathways pertinent to substance and energy metabolism, and engaging in non-enzymatic oxidative decarboxylation reactions during ammonia detoxification (Figure 11). Notwithstanding these findings, further investigation is warranted to reveal the molecular dynamics of AKG, particularly its role in signaling and the potential epigenetic regulation of genes involved in ammonia detoxification.

## Data Availability

Data are contained within the article and Supplementary Materials.

## Supplementary Materials

The following supporting information can be downloaded at https://www.mdpi.com/article/10.3390/molecules29071425/s1. Figure S1: Effects of different concentrations of NH_4_Cl treatment on poplar *Nanlin 895* for two weeks; Figure S2: Effects of foliar spraying of different concentrations of AKG on poplar *Nanlin 895* for 20 days; Figure S3: The entire native PAGE gel images showing activities of the carbon and nitrogen metabolism enzymes that were compatible with Figure 4. Table S1: Quantitative real-time PCR (qPCR) primer sequences used in this study.

## References

[1] Gong J., Zhang Z., Wang B., Shi J., Zhang W., Dong Q., Song L., Li Y., Liu Y. (2022). N addition rebalances the carbon and nitrogen metabolisms of *Leymus chinensis* through leaf N investment. Plant Physiol. Biochem..

[2] Insausti M., Timmis R., Kinnersley R., Rufino M.C. (2020). Advances in sensing ammonia from agricultural sources. Sci. Total Environ..

[3] Sutton M.A., Erisman W., Leip A., van Grinsven H., Winiwarter W. (2011). Too much of a good thing. Nature.

[4] Su S., Zhou Y., Qin J.G., Wang W., Yao W., Song L. (2012). Physiological responses of *Egeria densa* to high ammonium concentration and nitrogen deficiency. Chemosphere.

[5] Schjoerring J.K., Husted S., Mäck G., Nielsen K.H., Finnemann J., Mattsson M. (2000). Physiological regulation of plant-atmosphere ammonia exchange. Plant Soil.

[6] Hessini K. (2022). Nitrogen form differently modulates growth, metabolite profile, and antioxidant and nitrogen metabolism activities in roots of *Spartina alterniflora* in response to increasing salinity. Plant Physiol. Biochem..

[7] Zhou Q., Gao J., Zhang R., Zhang R. (2017). Ammonia stress on nitrogen metabolism in tolerant aquatic plant—*Myriophyllum aquaticum*. Ecotoxicol. Environ. Saf..

[8] Vega-Mas I., Perez-Delgado C.M., Marino D., Fuertes-Mendiza’bal T. (2017). Elevated CO_2_ induces root defensive mechanisms in tomato plants when dealing with ammonium toxicity. Plant Cell Physiol..

[9] Ariz I., Asensio A.C., Zamarreño A.M., García-Mina J.M., Aparicio-Tejo P.M., Moran J.F. (2013). Changes in the C/N balance caused by increasing external ammonium concentrations are driven by carbon and energy availabilities during ammonium nutrition in pea plants: The key roles of asparagine synthetase and anaplerotic enzymes. Physiol. Plant..

[10] Ten Hoopen F., Cuin T.A., Pedas P., Hegelund J.N., Shabala S. (2010). Competition between uptake of ammonium and potassium in barley and Arabidopsis roots: Molecular mechanisms and physiological consequences. J. Exp. Bot..

[11] Coskun D., Britto D.T., Li M., Becker A. (2013). Rapid ammonia gas transport accounts for futile transmembrane cycling under NH_3_/NH_4_^+^ toxicity in plant roots. Plant Physiol..

[12] Britto D.T., Siddiqi M.Y., Glass A.D.M., Kronzucker H.J. (2001). Futile transmembrane NH_4_^+^ cycling: A cellular hypothesis to explain ammonium toxicity in plants. Proc. Natl. Acad. Sci. USA.

[13] Wu N., Yang M., Gaur U., Xu H., Yao Y., Li D. (2016). Alpha-ketoglutarate: Physiological functions and applications. Biomol. Ther..

[14] Zhang C., Zhou C., Burnap R.L., Peng L. (2018). Carbon/Nitrogen Metabolic Balance: Lessons from Cyanobacteria. Trends Plant Sci..

[15] Abla H., Sollazzo M., Gasparre G., Iommarini L., Porcelli A.M. (2020). The multifaceted contribution of α-ketoglutarate to tumor progression: An opportunity to exploit?. Semin. Cell Dev. Biol..

[16] Chin R.M., Fu X., Pai M.Y., Vergnes L., Hwang H., Diep S., Lomenick B., Meli V.S., Monsalve G.C., Whelan S.A. (2014). The metabolite alpha-ketoglutarate extends lifespan by inhibiting the ATP synthase and TOR. Nature.

[17] Velvizhi S., Dakshayani K.B., Subramanian P. (2002). Effects of α-ketoglutarate on antioxidants and lipid peroxidation products in rats treated with ammonium acetate. Nutrition.

[18] Naeini S.H., Mavaddatiyan L., Kalkhoran Z.R., Taherkhani S., Talkhabi M. (2023). Alpha-ketoglutarate as a potent regulator for lifespan and healthspan: Evidences and perspectives. Exp. Gerontol..

[19] Gui P., Chen X., Liao Z., Wang L. (2016). Effect of organic carbon on carbon and nitrogen metabolism and the growth of water spanich as affected by nitrogen levels. Acta Pedol. Sin..

[20] Fu X., Gui R., Li W., Gao Z., Ashraf U., Tan J., Ye Q., Chen J., Xie H., Mo Z. (2021). Nitrogen and α-ketoglutaric acid application modulate grain yield, aroma, nutrient uptake and physiological attributes in fragrant rice. J. Plant Growth Regul..

[21] Magalhães J.R., Huber D.M., Tsai C.Y. (1992). Evidence of increased 15N-ammonium assimilation in tomato plants with exogenous α-ketoglutarate. Plant Sci..

[22] Singh M., Singh P., Prasad S.M. (2022). α-Ketoglutarate enhanced *Solanum melongena* L. growth: Acceleration of nitrogen assimilating enzymes and antioxidant system under arsenate toxicity. J. Plant Growth Regul..

[23] Alamri S., Alsubaie Q.D., Al-Amri A.A., Al-Munqedi B., Ali H.M. (2021). Priming of tomato seedlings with 2-oxoglutarate induces arsenic toxicity alleviatory responses by involving endogenous nitric oxide. Physiol. Plant..

[24] Lei S., Huang B. (2022). Metabolic regulation of α-Ketoglutarate associated with heat tolerance in perennial ryegrass. Plant Physiol. Biochem. J..

[25] Liu S., He L., Yao K. (2018). The antioxidative function of alpha-ketoglutarate and its applications. BioMed Res. Int..

[26] Gai Z., Liu J., Cai L., Zhang J., Liu L. (2022). Foliar application of alpha-ketoglutarate plus nitrogen improves drought resistance in soybean (*Glycine max* L. Merr.). Sci. Rep..

[27] Gai Z., Zhang M., Zhang P., Zhang J., Liu J., Cai L., Yang X., Zhang N., Yan Z., Liu L. (2023). 2-Oxoglutarate contributes to the effect of foliar nitrogen on enhancing drought tolerance during flowering and grain yield of soybean. Sci. Rep..

[28] Zhang C., Chen J., Zhuang S., Feng Z., Fan J. (2023). Functional characterization of PsAMT1.1 from *Populus simonii* in ammonium transport and its role in nitrogen uptake and metabolism. Environ. Exp. Bot..

[29] Thakur A.K., Kumar P., Parmar N., Shandil R.K., Aggarwal G., Gaur A., Srivastava D.K. (2021). Achievements and prospects of genetic engineering in poplar: A review. New For..

[30] Rennenberg H., Dannenmann M. (2015). Nitrogen nutrition of trees in temperate forests-the significance of nitrogen availability in the pedosphere and atmosphere. Forests.

[31] Krupa S.V. (2003). Effects of atmospheric ammonia (NH_3_) on terrestrial vegetation: A review. Environ. Pollut..

[32] Du Y.D., Yuan X.Y., Feng Z.Z. (2023). Effects of different nitrogen forms on photosynthesis characteristics and growth of poplar. Chin. J. Plant Ecol..

[33] Guo H., Wang H., Liu Q., An H., Liu C., Xia X., Yin W. (2017). ^15^N-labeled ammonium nitrogen uptake and physiological responses of poplar exposed to PM_2.5_ particles. Environ. Sci. Pollut. Res..

[34] Esteban R., Ariz I., Cruz C., Moran J.F. (2016). Review: Mechanisms of ammonium toxicity and the quest for tolerance. Plant Sci..

[35] Liang C., Zhang Y., Cheng S., Osorio S., Sun Y., Fernie A.R., Cheung C.Y.M., Lim B.L. (2015). Impacts of high ATP supply from chloroplasts and mitochondria on the leaf metabolism of *Arabidopsis thaliana*. Front. Plant Sci..

[36] Noguchi K., Yoshida K. (2008). Interaction between photosynthesis and respiration in illuminated leaves. Mitochondrion.

[37] Legendre F., MacLean A., Appanna V.P., Appanna V.D. (2020). Biochemical pathways to α-ketoglutarate, a multi-faceted metabolite. World J. Microbiol. Biotechnol..

[38] Huergo L.F., Dixon R. (2015). The emergence of 2-Oxoglutarate as a master regulator metabolite. Microbiol. Mol. Biol. Rev..

[39] Reitzer L. (2003). Nitrogen assimilation and global regulation in *Escherichia coli*. Annu. Rev. Microbiol..

[40] Harper C.J., Hayward D., Kidd M., Wiid I., van Helden P. (2010). Glutamate dehydrogenase and glutamine synthetase are regulated in response to nitrogen availability in *Myocbacterium smegmatis*. BMC Microbiol..

[41] Chellamuthu V.R., Ermilova E., Lapina T., Lüddecke J., Minaeva E., Herrmann C., Hartmann M.D., Forchhammer K. (2014). A widespread glutamine-sensing mechanism in the plant kingdom. Cell.

[42] Jiang P., Ninfa A.J. (2009). α-ketoglutarate controls the ability of the *Escherichia coli* PII signal transduction protein to regulate the activities of NRII (NtrB) but does not control the binding of PII to NRII. Biochemistry.

[43] Pérez-Díaz J., Batista-Silva W., Almada R., Medeiros D.B. (2021). *Prunus hexokinase 3* genes alter primary c-metabolism and promote drought and salt stress tolerance in Arabidopsis transgenic plants. Sci. Rep..

[44] Sun Q., Liang W.W., Jia L., Wang Z.Q., Lin T.B. (2014). Effect of α-ketoglutaric acid on yield-related traits in winter wheat under low water and nitrogen. J. Anhui Agric. Sci..

[45] Yuan Y., Ou J., Wang Z., Zhang C., Zhou Z., Lin Q. (2007). Regulation of carbon and nitrogen metabolisms in rice roots by 2-oxoglutarate at the level of hexokinase. Physiol. Plant..

[46] Suarez M.F., Avila C., Gallardo F., Canton F.R. (2002). Molecular and enzymatic analysis of ammonium assimilation in woody plants. J. Exp. Bot..

[47] Mullen A.R., Hu Z., Shi X., Jiang L., Boroughs L.K., Kovacs Z., Boriack R., Rakheja D., Sullivan L.B., Linehan W.M. (2014). Oxidation of alpha-ketoglutarate is required for reductive carboxylation in cancer cells with mitochondrial defects. Cell Rep..

[48] Brugière N., Dubois F., Limami A.M., Lelandais M., Roux Y., Sangwan R.S., Hire B. (1999). Glutamine synthetase in the phloem plays a major role in controlling proline production. Plant Cell.

[49] Martínez-Reyes I., Chandel N.S. (2020). Mitochondrial TCA cycle metabolites control physiology and disease. Nat. Commun..

[50] Lin W., Jacobs-Wagner C. (2022). Connecting single-cell ATP dynamics to overflow metabolism, cell growth, and the cell cycle in *Escherichia coli*. Curr. Biol..

[51] Zhang Z., Hu M., Yun Z., Wang J., Feng G., Gao Z., Shi X., Jiang Y. (2017). Effect of tea seed oil treatment on browning of litchi fruit in relation to energy status and metabolism. Postharvest Biol. Technol. J..

[52] Zhou S., Zhang M., Wang P. (2021). Response of plant plasma membrane H^+^-ATPase to environmental stress factors: A review. Chin. J. Appl. Environ. Biol..

[53] Erdal S. (2019). Melatonin promotes plant growth by maintaining integration and coordination between carbon and nitrogen metabolisms. Plant Cell Rep..

[54] Rosenberger C.L., Chen J. (2018). To grow or not to grow: TOR and SnRK2 coordinate growth and stress response in Arabidopsis. Mol. Cell.

[55] Harrison A., Pierzynowski S.G. (2008). Biological effects of 2-oxoglutarate with particular emphasis on the regulation of protein, mineral and lipid absorption/metabolism, muscle performance, kidney function, bone formation and cancerogenesis, all viewed from a healthy ageing perspective state. J. Physiol. Pharmacol..

[56] Tcherkez G., Carroll A., Abadie C., Mainguet S., Davanture M., Zivy M. (2020). Protein synthesis increases with photosynthesis via the stimulation of translation initiation. Plant Sci..

[57] Zdzisińska B., Żurek A., Kandefer-Szerszeń M. (2017). Alpha-ketoglutarate as a molecule with pleiotropic activity: Well-known and novel possibilities of therapeutic use. Arch. Immunol. Ther. Exp..

[58] Chen Q., Vazquez E.J., Moghaddas S., Hoppel C.L. (2003). Production of reactive oxygen species by mitochondria. J. Biol. Chem..

[59] Puntel R.L., Roos D.H., Grotto D., Garcia S.C., Nogueira C.W., Batista Teixeira Rocha J. (2007). Antioxidant properties of Krebs cycle intermediates against malonate pro-oxidant activity in vitro: A comparative study using the colorimetric method and HPLC analysis to determine malondialdehyde in rat brain homogenates. Life Sci..

[60] Hu L., Zhang Z., Xiang Z., Yang Z. (2016). Exogenous application of citric acid ameliorates the adverse effect of heat stress in tall fescue (*Lolium arundinaceum*). Front. Plant Sci..

[61] Han M., Xu M.Y., Wang S.Z., Wu L.D., Sun S.Y., Su T. (2022). Effects of exogenous L-Glutamine as a sole nitrogen source on physiological characteristics and nitrogen use efficiency of poplar. Plant Physiol. Biochem..

[62] Gong L., Zhang S., Chen D., Liu K., Lu J. (2018). Response of biofilms-leaves of two submerged macrophytes to high ammonium. Chemosphere.

[63] Li L., Gu W., Li J., Li C., Xie T., Qu D., Meng Y., Li C., Wei S. (2018). Exogenously applied spermidine alleviates photosynthetic inhibition under drought stress in maize (*Zea mays* L.) seedlings associated with changes in endogenous polyamines and phytohormones. Plant Physiol. Biochem..

[64] Su T., Han M., Min J., Cao D., Zhai G., Zhou H., Li N., Li M. (2019). Genome-wide characterization of *AspATs* in *Populus*: Gene expression variation and enzyme activities in response to nitrogen perturbations. Forests.

[65] Singh R., Chénier D., Bériault R., Mailloux R., Hamel R.D., Appanna V.D. (2005). Blue native polyacrylamide gel electrophoresis and the monitoring of malate- and oxaloacetate-producing enzymes. J. Biochem. Biophys. Methods.

[66] Lin Y., Lin Y., Lin H., Ritenour M.A., Shi J., Zhang S., Chen Y., Wang H. (2017). Hydrogen peroxide-induced pericarp browning of harvested longan fruit in association with energy metabolism. Food Chem..

[67] Patterson K., Cakmak T., Cooper A., Lager I., Rasmusson A.G., Escobar M.A. (2010). Distinct signalling pathways and transcriptome response signatures differentiate ammonium- and nitrate-supplied plants. Plant. Cell Environ..

[68] Bräutigam A., Gagneul D., Weber A.P.M. (2007). High-throughput colorimetric method for the parallel assay of glyoxylic acid and ammonium in a single extract. Anal. Biochem..

[69] Liao Y., Cui R., Yuan T., Xie Y., Gao Y. (2019). Cysteine and methionine contribute differentially to regulate alternative oxidase in leaves of poplar (*Populus deltoides* x *Populus euramericana* ‘Nanlin 895’) seedlings exposed to different salinity. J. Plant Physiol..

[70] Zhang T., Cao Y., Chen Y., Liu G. (2015). Non-structural carbohydrate dynamics in *Robinia pseudoacacia* saplings under three levels of continuous drought stress. Trees.

[71] Patterson B.D., Payne L.A., Chen Y.-Z., Graham D. (1984). An inhibitor of Catalase induced by cold in chilling-sensitive plants. Plant Physiol..

[72] Livak K.J., Schmittgen T.D. (2001). Analysis of relative gene expression data using real-time quantitative PCR and the 2^−ΔΔCT^ Method. Methods.

