# Supplementary material for: Regulating Effect of Exogenous α-Ketoglutarate on Ammonium Assimilation in Poplar
Source: Molecules. 2024 Mar 22;29(7):1425. doi: 10.3390/molecules29071425 (PMC11012726; doi:10.3390/molecules29071425)
Supplement: Supplementary file 1 [file molecules-29-01425-s001.zip › molecules-2916488-supplementary.pdf]

Table S1 Quantitative Real-time PCR (qPCR) primers design information

Gene ID	Forward primer	Reverse primer
PtrGlnB	GTGAACGTGGAGAGAAGGCT	ATCTTGAGCTGCGCATGTTG
PtrGS1	CAAAACCCAAGAGTAAAAAGGTCC	CCAGCAAGAGTTTTATTTAGATTAG
PtrGS2	GGAGCATCACTTGGATCTAGATGG	CAAAACCCAAGAGTAAAAAGGTCC
PtrAMPK	GTTCCAAGCTCACCTTCCAC	CAACGCTTGCCTCATTTTGC
PtrSNRK2.9	GGGAGCATGGATCTTGATCA	AACATCGCCCCAGTTTTGTA
PtrSNRK2.10	ATTGACAGCAGTGGGGAGAT	CCCATGCTGTTGCCTATTCT
PtrSNRK2.12	CACCCCAAGTCTCAACCATT	CCCCGCTCTCTTACAGTCAC
PtrTOR1	GTCATGTCCCTGCTGTTGTG	GACAACCACAGCACGTTCAT
PtrTOR2	CGTGATTTGGAGCTTGCTGT	TCGTCTTGGCGCAAATCTTC
PtrHXK3	TACAGCGTGGATGGCGTAAA	ACGGGCGACTTGAGAAGAAA
PtrICDH3	CCTCTGTATTGGTGTGCCCT	CCAGACTCCACAGCTCCAAC
PtrCOX2	CACTGGTTATGGCGGAACTG	GCACTTTCTCCTGGCTTCAC
PtrAOX2	GCATGGTGGGGTATCTCGAG	AACAGCCACAACAACATCGC
PtrAtpb-1	TTTAGCCCCTTATCGCCGTG	GTACGTTCACCTACTCCGCC
PtrAtpb-2	TCGTGGTGTGCAGAAGGTTC	AACCGTCAGCTTGTCGTCTT
PtrNDC	CGCTGCTTCTCCTAGTCCAG	AAGCCTCCAAATCCACCACC
PtUBIC	GATTACCCGGAGAAGCCACC	GTTGTGTGGTGCTGTCATCT

PtrGlnB: Nitrogen regulatory protein P_{II}; *PtrGS*: Glutamine synthetase; *PtrAMPK*: Adenosine monophosphate-activated protein kinase; *PtrSNRK*: Sucrose nonfermenting 1-related kinase; *PtrTOR*: Target of rapamycin; *PtrHXK*: Hexokinase; *PtrICDH*: Isocitrate dehydrogenase; *PtrCOX*: Cytochrome c oxidase; *PtrAOX:* Alternative oxidase; *PtrAtpb*: β subunit of ATP synthase; *PtrNDC*: NADH ubiquinone oxidoreductase.

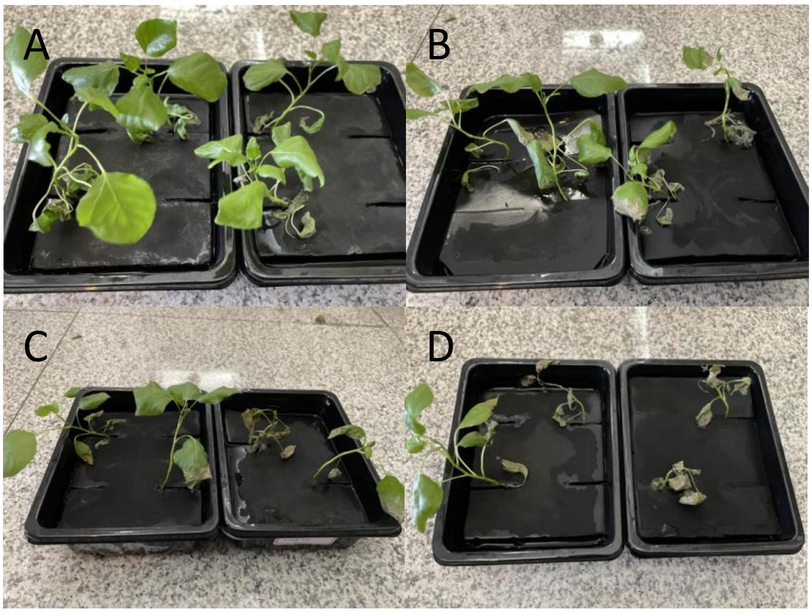

Fig. S1. Effects of different concentrations of NH_4Cl treatment on poplar *Nanlin 895* for 2 weeks. (A) 1mM NH_4Cl, (B) 3 mM NH_4Cl, (C) 5 mM NH_4Cl, (D) 10 mM NH_4Cl.

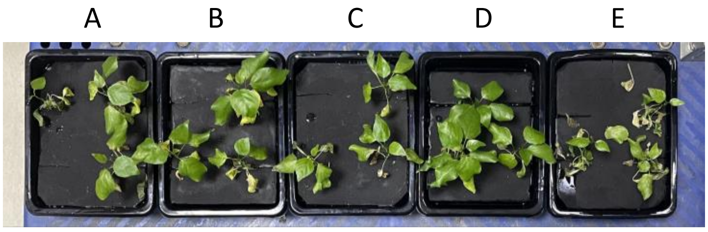

Fig. S2. Effects of foliar spraying of different concentrations of AKG on poplar *Nanlin 895* for 20 days. (A) 0 mM AKG, (B) 0.05 mM AKG, (C) 0.5 mM AKG, (D) 5 mM AKG, (E) 10 mM AKG.

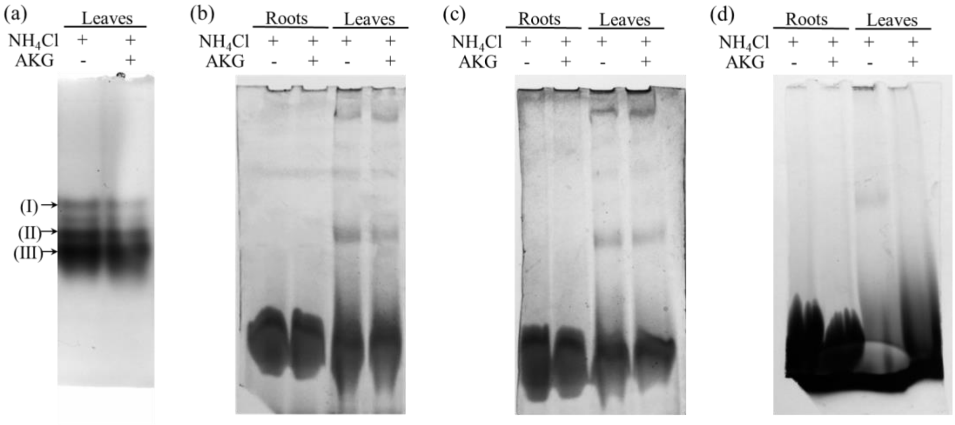

Fig S3. The entire native PAGE gel images showing activities of the carbon and nitrogen metabolism enzymes that were compatible with Fig 4. (a) Aspartate aminotransferase (AspAT) activity. (I) Mitochondrial AspAT; (II) Cytoplasmic AspAT; (III) Chloroplast AspAT. (b) NADP-dependent isocitrate dehydrogenase (ICDH-NADP) activity. (c) NAD-dependent isocitrate dehydrogenase (ICDH-NAD) activity. (d) NADP-dependent malic enzyme (ME-NADP) activity.
